# Influence of body mass index on incidence and prognosis of acute myeloid leukemia and acute promyelocytic leukemia: A meta-analysis

**DOI:** 10.1038/s41598-017-18278-x

**Published:** 2017-12-21

**Authors:** Shufen Li, Li Chen, Wen Jin, Xuefei Ma, Yunlin Ma, Fangyi Dong, Hongming Zhu, Junmin Li, Kankan Wang

**Affiliations:** 1 State Key Laboratory of Medical Genomics and Shanghai Institute of Hematology, Ruijin Hospital, Shanghai Jiao Tong University School of Medicine, Shanghai, 200025 China; 2 Department of Hematology, Ruijin Hospital, Shanghai Jiao Tong University School of Medicine, Shanghai, 200025 China; 3 State Key Laboratory of Medical Genomics, Institute of Health Sciences, Shanghai Institutes for Biological Sciences, Chinese Academy of Sciences & Shanghai Jiao Tong University School of Medicine, University of Chinese Academy of Sciences, Shanghai, China

## Abstract

Previous studies have demonstrated an association between high body mass index (BMI) and acute myeloid leukemias (AML), particularly acute promyelocytic leukemia (APL). However, the effect of obesity and overweight on the incidence of AML is not supported by all studies, and the relationship between obesity and prognosis of AML and APL has not been established. Thus, we conducted a meta-analysis to determine the role of BMI on the risk and clinical outcome of AML, including APL. Twenty-six eligible studies enrolling 12,971 AML (including 866 APL) patients were retrieved and analyzed. Overweight and obesity was associated with an increased incidence of AML (relative risk [RR], 1.23; 95% confidence interval [CI], 1.12–1.35; P < 0.001). High BMI did not significantly affect overall survival (OS) (hazard ratio [HR], 0.97; 95% CI, 0.92–1.03; P = 0.323) or disease-free survival (HR, 0.98; 95% CI, 0.88–1.10; P = 0.755) in patients with non-APL AML. By contrast, APL patients with high BMI had shorter OS (HR, 1.77; 95% CI, 1.26–2.48; P = 0.001) and a higher risk of differentiation syndrome (HR, 1.53; 95% CI, 1.03–2.27, P = 0.04). Overall, our findings suggest that patients with overweight or obesity have a higher incidence of AML, and high BMI is a predictor of adverse clinical outcomes in APL.

## Introduction

The prevalence of obesity has been increasing worldwide over the past decades. According to World Health Organization (WHO), overweight and obesity are defined by a body mass index (BMI) of 25–29.9 kg/m^2^ and ≥30 kg/m^2^, respectively. Currently, the proportion of overweight or obesity in adults has increased to approximately 40% worldwide^1^. It has been reported that overweight or obesity is associated with over 20 types of cancer, such as cancers of the breast, colon, uterus, gallbladder, and cervix, as well as leukemia^2,3^.

Leukemia is a clonal hematopoietic disorder characterized by the aggressive proliferation of immature hematopoietic progenitor cells arrested at an early stage of differentiation. Acute myeloid leukemia (AML) is one of the most common myeloid malignancies in adults^4^. Although much effort has been devoted to studying this disease, the etiology and pathophysiology of AML are still poorly understood and the outcome remains unsatisfactory. Epidemiologic observations have suggested that obesity is a risk factor for AML^5–7^. However, obesity did not affect risk for AML in several studies^8–10^, and Wong *et al*. even reported that obesity is protective against AML^11^. Overweight may have a similar effect on the incidence of AML, although this relationship is less certain^6,8,12^. The most recent meta-analysis on this topic was performed in 2012 and suggested that obesity, but not overweight, increases incidence of AML^7^. However, the number of patients with AML was limited because this meta-analysis included all types of leukemia and only included five studies on AML. Since 2012, five new studies, including two large multinational survey programs, have been published^5,6,10,12,13^. Thus, including these new studies in an updated analysis is needed to comprehensively evaluate the association between BMI and risk for AML.

In addition to the incidence of AML, the association between high BMI and the prognosis of AML is unclear due to the lack of a meta-analysis, although several studies have investigated this issue^14–16^. Reports on the prognostic value of obesity for AML are somewhat inconsistent. Several studies have found that obesity adversely affects the clinical outcome of AML^17^ whereas others have failed to confirm such an association^15,18,19^.

Notably, several studies have shown that the percentage of obese patients with acute promyelocytic leukemia (APL), a unique subtype of AML, is higher than that observed in patients with non-APL AML^15,20,21^. APL generally carries the PML/RARα fusion protein linking the retinoic acid receptor alpha gene (RARα) on chromosome 17 with the *PML* gene on chromosome 15. Historically, APL was one of the most fatal forms of acute leukemia but currently has a cure rate of approximately 80–85% with the introduction of all-*trans* retinoic acid (ATRA) and arsenic trioxide (ATO)^15,22^. Emerging studies have demonstrated that obesity has adverse effects on the treatment outcome of APL^15,23^. However, to our knowledge, a quantitative analysis that evaluates this association with APL is not available.

With the aim of evaluating the prognostic value of obesity in patients with AML (including APL), we conducted the first comprehensive meta-analysis to clarify the potential association between AML, particularly APL, with overweight and obesity as defined by BMI. Another objective of our study was to investigate the association between obesity and the incidence of AML.

## Methods

### BMI calculation and classification

BMI was calculated by dividing weight in kilograms by the square of height in meters (weight [kg]/height [m^2^]). According to the criteria from the World Health Organization, BMI was categorized as underweight (<18.5 kg/m^2^), normal (18.5–25 kg/m^2^), overweight (25–29.9 kg/m^2^), and obese (≥30 kg/m^2^). Most of the studies used 25 and 30 kg/m^2^ as the thresholds for overweight and obesity, respectively. Studies that did not meet these criteria^11,13,24^ were eligible for analysis of the association based on high BMI but were excluded from the overweight and obese subgroup analysis. Lin *et al*. used body weight (BW) ≥130% ideal body weight (IBW) as the criteria to define obesity^25^.

### Literature search

A literature search (last search updated to Dec. 4, 2016) was conducted in PubMed, EMBASE, Web of Science and the Cochrane Library for articles assessing the effect of overweight and obesity on the incidence and clinical outcome of AML using the keywords as follows: (obesity OR overweight OR body mass OR BMI OR body weight OR anthropometric) AND leukemia. References lists from the retrieved studies were also examined. The results were limited to peer-reviewed English language studies.

### Eligibility criteria

The studies were considered eligible if they reported the effect of overweight and obesity on the incidence or clinical outcome of patients with AML and provided sufficient data to determine an estimate of relative risk (RR) or hazard ratio (HR) and a 95% confidence interval (CI). Odd ratios (OR) were converted to RRs using methods reported by Zhang *et al*.^26^. When the patient populations overlapped between studies, only the most recent or most complete publication was included to avoid duplications.

### Data extraction and quality assessment

The data extracted for our meta-analysis included the first author’s name, year of publication, country, number of patients analyzed, enrollment period, source of the cohort, adjustments, and other relevant data stratified by BMI. Quality assessment of eligible papers was conducted according to the Newcastle-Ottawa Scale (NOS)^7^. The NOS score includes an assessment of subject selection (four points), comparability of groups (two points), and exposure or clinical outcome (three points). The maximum score is 9 and the scores of eligible papers included in our meta-analysis is ranged from 5 to 9 (Supplementary Table S1).

### Statistical analysis

RR or HR from each article was extracted directly from the original reports or calculated using the method reported by Tierney *et al*.^27^. The potential heterogeneity across studies was evaluated using the Cochran’s Q-test and expressed using the I^2^ index. The pooled results for RR and HR were calculated by the fixed-effects model (I^2^ ≤ 50%) or the random-effects model (I^2^ > 50%). Publication bias was evaluated by the funnel plot and Egger’s and Begg’s tests. The effect of publication bias on the pooled findings was evaluated by trim-and-fill analyses. The stability of the pooled findings was confirmed by one-way sensitivity and subgroup analyses. All statistical analyses were conducted using STATA version 11.0.

## Results

### Identification of relevant studies

**Figure 1:**
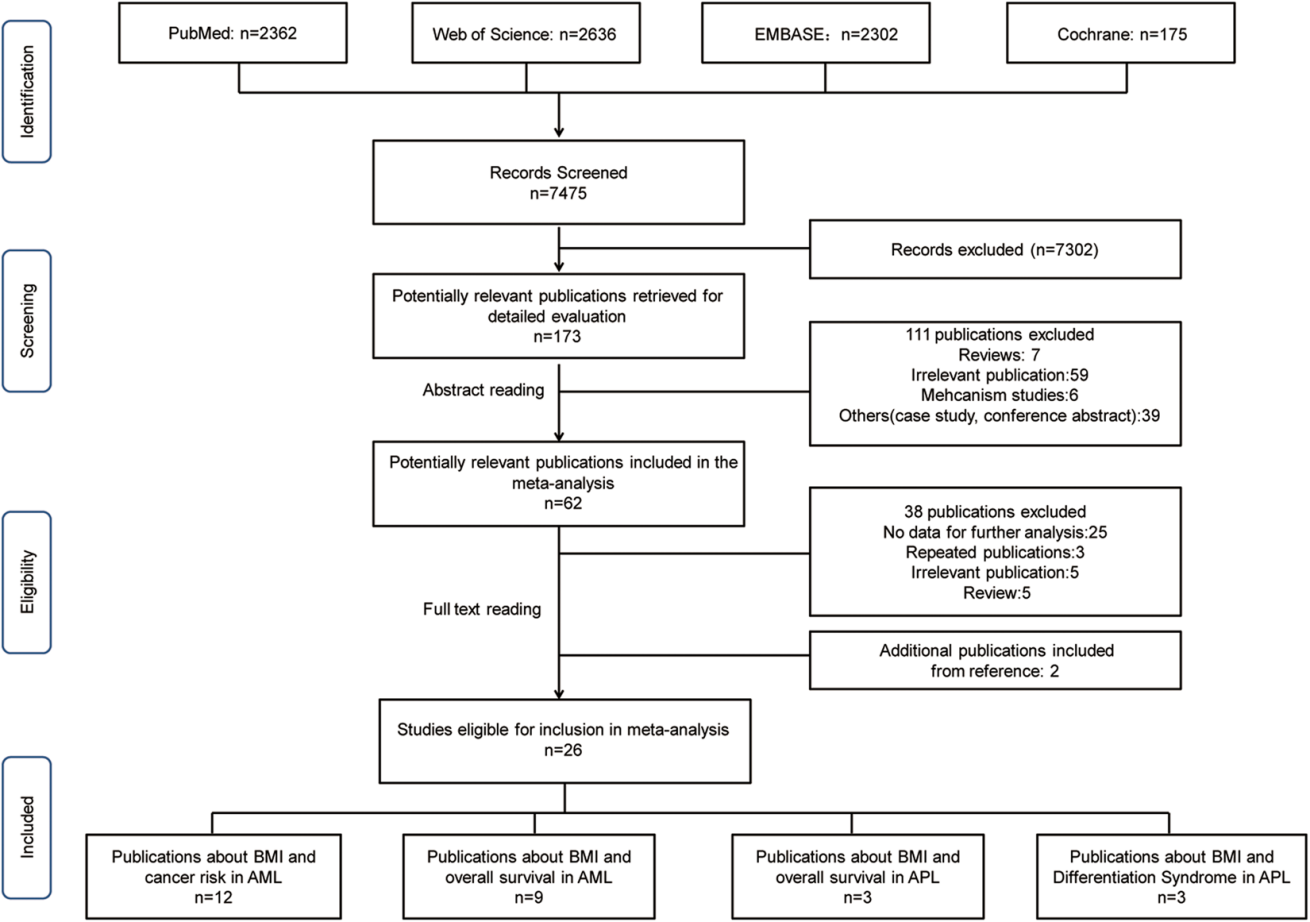
Results of the search strategy. BMI, body mass index; AML, acute myeloid leukemia; APL, acute promyelocytic leukemia.

A total of 7,475 studies were retrieved from the preliminary literature search (PubMed:2,362, EMBASE:2,302, Web of Science:2,636, and the Cochrane library:175), and an additional two studies were identified from a review of citations. 173 potential relevant publications were retrieved for detailed evaluation. Of these publications, 111 articles were excluded after reading the title and abstract because they were irrelevant to our meta-analysis, or because they were mechanistic studies, case reports, conference abstracts, or reviews. An additional 38 publications were then excluded because they were irrelevant to AML, provided no data for further analysis, or were duplicate publications or reviews. Finally, 26 studies were included in our meta-analysis (Fig. 1, Table 1), including 12 papers about relative risk (RR) with 6,724 AML patients^5,6,8–13,28–31^, 11 papers regarding AML survival with 5,505 non-APL AML patients and 538 APL patients^14–19,24,25,32–34^, and three papers on the subject of differentiation syndrome in APL (after excluding duplicate publications^32,35,36^) with 243 patients^23,37,38^.

**Table 1:** Characteristics of studies included in the meta-analysis.

First author, year	Country	Subtype (patient #)	Enrollment period	Source of the cohort	Adjustments
**Studies on relative risk**					
Samanic 2004	USA	AML (1894)	1969–1996	Veterans Affairs (VA) Hospitals	age, race and calendar-year
Ross 2004	USA	AML (74)	1986–2001	The Iowa Women’s Health Study	age and regular physical activity
Kasim 2005	Canada	AML (307)	1994–1997	Canadian National Enhanced Cancer Surveillance System	gender and pack-years of smoking
Samanic 2006	Sweden	AML (267)	1971–1992	The Population- based Swedish Cancer Registry, The Nationwide Mortality Registry, the Migration Register	age and smoking status
Engeland 2007	Norway	AML (1374)	1963–2001	Norwegian Nationwide Screening Program	birth cohort and age
Wong 2009	China	non-APL AML (598)APL (124)	2003–2007	Twenty-nine Hospitals in Shanghai	gender, age
Soderberg 2009	Sweden, Finland	AML (66)	1961–2002 Sweden1975–2004 Finland	The Swedish Twin Registry and one Finnish Twin Cohort	age, sex, country, alcohol intake, education, smoking, diabetes and exercise
Strom 2012	USA	AML (638)	2003–2007	The University of Texas M. D. Anderson Cancer Center	education and family history of hematopoietic cancer
Nagel 2012	Norway, Sweden, Austria	AML (231)	1988–2002 Norway1972–2003Sweden1974–2005 Austria	The Metabolic Syndrome and Cancer Project (Me-Can)	age, smoking status
Murphy 2013	UK	AML (578)	1996–2001	The United Kingdom National Breast Cancer Screening Programme	height, alcohol consumption, smoking and socioeconomic status
Hosnijeh 2013	Denmark, France, Greece, Germany, Italy, Netherlands, Norway, Spain, Sweden, UK	AML (153)	1992–2000	European Prospective Investigation into Cancer and Nutrition (EPIC)	physical activity, educational level, smoking status, alcohol intake, history of diabetes and family history of cancer
Poynter 2016	USA	AML (420)	2005–2009	Minnesota Cancer Surveillance System	age, income, physical activity, and exposure to chemotherapy or benzene
**Studies on overall survival**					
Jeddi 2010	**Tunisia**	APL (39)	2004–2008	Aziza Othmana University Hospital	age, sex, baseline WBC, serum creatinine, platelet count and immune phenotyping
Lee 2012	USA	non-APL AML (329)	1990–2008	Roswell Park Cancer Institute	age, gender, AML presentation, WBC, smoking history, treatment decade and karyotype
Medeiros 2012	USA	non-APL AML (1974)	1980s–2000s	Southwest Oncology Group (Trial S8600, S9031, S9126, S9333, S9500, S9617, S9918, and S0106)	age, gender, performance status, karyotype, WBC, platelet and peripheral blast counts
Lin 2013	USA	non-APL AML (63)	2006–2010	University of Washington Medical Center	prior malignancy, FLT3-ITD and NPM-1 status
Wenzell 2013	USA	non-APL AML (247)	2002–2009	Cleveland Clinic	age, sex, WBC, cytogenetic risk, etiology and bacteremia
Brunner 2013	USA	non-APL AML (97)	1992–2011	Massachusetts General Hospital	a history of CAD or diabetes, patient gender and race, patient cytogenetics
Kempf 2014^a^	France	non-APL AML (233)	2003–2013	Saint-Antoine hospital	age, gender and cytogenetic
Wang 2015	China	APL (53)	2004–2010	Institute of Hematology and Blood Diseases Hospital	not available
Finn 2015	USA	non-APL AML (295)	1995–2012	Mayo Clinic in Florida and Arizona	not available
Castillo 2016^a^	USA	APL (446) non-APL AML (1648)	APL (1999–2005)AML (1993–2010)	APL from CALGB9710, non-APL AML from CALGB9621, 10503 and 19808	age, sex, performance status, race, ethnicity, treatment and WBC
Tavitian 2016^a^	France	non-APL AML (619)	2004–2012	Toulouse University Hospital	age, WBC, AML status and ECOG performance status
**Studies on differentiation syndrome**					
Jeddi 2010	Tunisia	APL (39)	2004–2008	Aziza Othmana University Hospital	age, sex, WBC, serum creatinine, platelet count and immunophenotyping
Breccia 2012	Rome	APL (144)	1993–2010	Sapienza University of Rome	age, sex, FAB classification, transcript type, WBC, platelet count and hemoglobin level
Leblebjian 2013	USA	APL (60)	2004–2010	Dana Farber/Brigham and Women’s Hospital Cancer Center (DF/BWHCC)	age, sex, WBC, percent blast count, serum creatinine, platelet count, uric acid, lactate dehydrogenase, albumin and type of chemotherapy used with ATRA

Abbreviations: AML, acute myeloid leukemia; APL, acute promyelocytic leukemia; DS, differentiation syndrome; OR, odds ratio; RR, relative ratio; OS, overall survival; WBC, white blood count.

^a^Containing disease-free survival (DFS) data.

### Relationship between high BMI and incidence of AML

**Figure 2:**
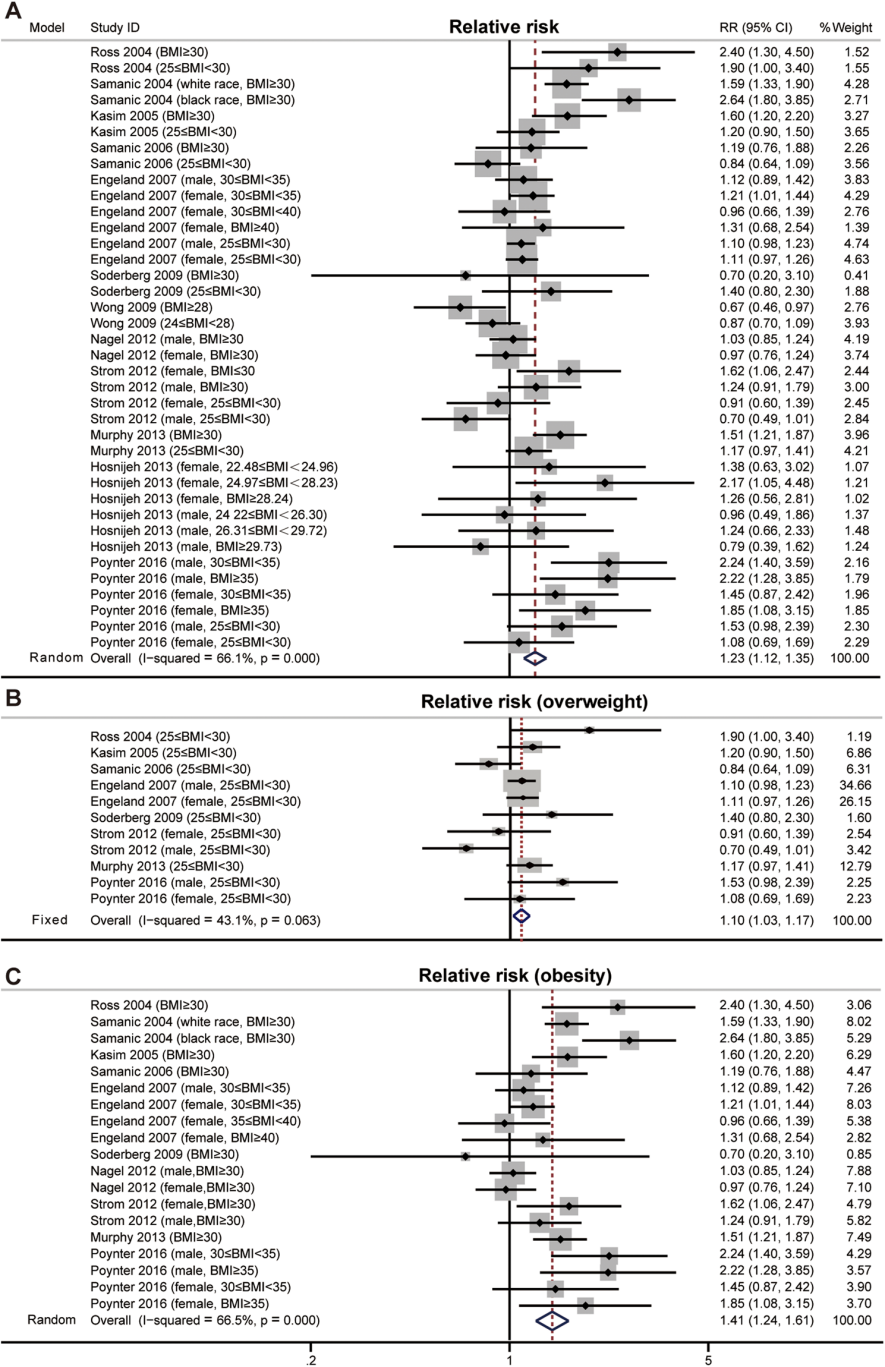
The effects of high BMI on the incidence of AML. (**A**) Meta-analysis of the relative risk (RR) of AML according to BMI with random-effects model. (**B**) Estimates of RR in overweight AML individuals with the fixed-effects model. (**C**) Estimates of RR in obese AML individuals with the random-effects model.

AML is one of the most common myeloid malignancies in adults. To explore the association between BMI and the incidence of AML, we analyzed 12 studies that reported on incidence estimates for AML according to BMI (Table 1). All ORs were transformed into RRs before pooled analysis. The pooled RR indicated that high BMI was associated with increased risk for AML (RR, 1.23; 95% CI, 1.12–1.35; P < 0.001; I^2^ = 66.0%; random effects; Fig. 2A). Although no significant publication bias was identified by Begg’s and Egger’s tests (P_Begg_ = 0.227; P_Egger_ = 0.156), the funnel plot suggested the possibility of missing studies (Supplementary Fig. S1A). After performing a trim-and-fill analysis (Supplementary Fig. S1B), we found that six studies might be missing. When these potentially missing studies were added to the analysis, the adjusted RR would be 1.14 (95% CI, 1.04–1.26, P = 0.008).

We further divided the patients into overweight and obesity subgroups. As shown in Fig. 2B and C, both overweight and obesity were associated with higher incidence of AML (for overweight, RR, 1.10; 95% CI, 1.03–1.17; P = 0.007; I^2^ = 43.1%; fixed effects; and for obesity, RR, 1.41; 95% CI, 1.24–1.61; P < 0.001; I^2^ = 66.5%; random effects). One-way sensitivity analysis further confirmed the stability of our results (Supplementary Fig. S1C).

### Relationship between high BMI and clinical outcome of AML

Next, we investigated the prognostic value of BMI for clinical outcomes of AML. Because treatment strategy and prognosis of APL and non-APL are significantly different, we analyzed their clinical outcomes separately.

#### No association between high BMI and clinical outcome of non-APL AML

**Figure 3:**
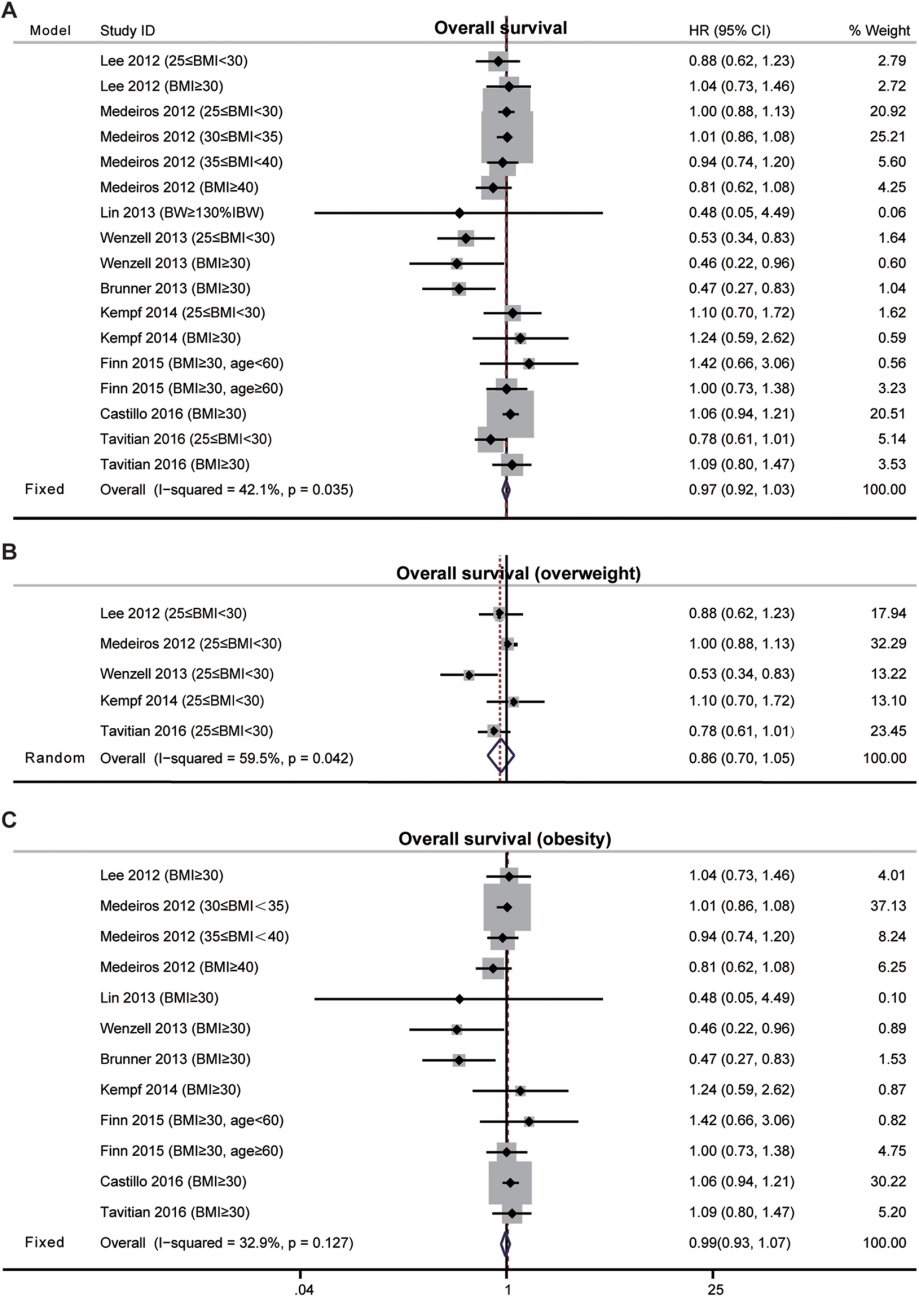
The effects of high BMI on the overall survival of non-APL AML patients. (**A**) Meta-analysis of the overall survival (OS) of AML according to BMI with the fixed-effects model. (**B**) Estimates of OS in overweight AML individuals with the random-effects model. (**C**) Estimates of OS in obese AML individuals with fixed-effects model.

Nine studies were included in the survival estimates for BMI in non-APL AML (Table 1). Our data suggested that high BMI did not significantly affect the survival of non-APL AML patients (HR, 0.97; 95% CI, 0.92–1.03; P = 0.323; I^2^ = 42.1%, fixed effects, Fig. 3A). No significant publication bias was identified by Begg’s and Egger’s tests (P_Begg_ = 0.434; P_Egger_ = 0.25). Although the funnel plot showed asymmetry (Supplementary Fig. S2A), no missing study were identified by the trim-and-fill analysis (Supplementary Fig. S2B).

We divided these patients into overweight and obese subgroups. Similarly, neither overweight nor obesity affected OS of patients with non-APL AML relative to patients of normal weight (for overweight, HR, 0.86; 95% CI, 0.70–1.05; P = 0.14; I^2^ = 59.5%; random effects; and for obesity, HR, 0.99; 95% CI, 0.93–1.07; P = 0.281; I^2^ = 32.9%; fixed effects; Fig. 3B,C). One-way sensitivity analysis further confirmed the stability of our pooled findings (Supplementary Fig. S2C).

**Figure 4:**
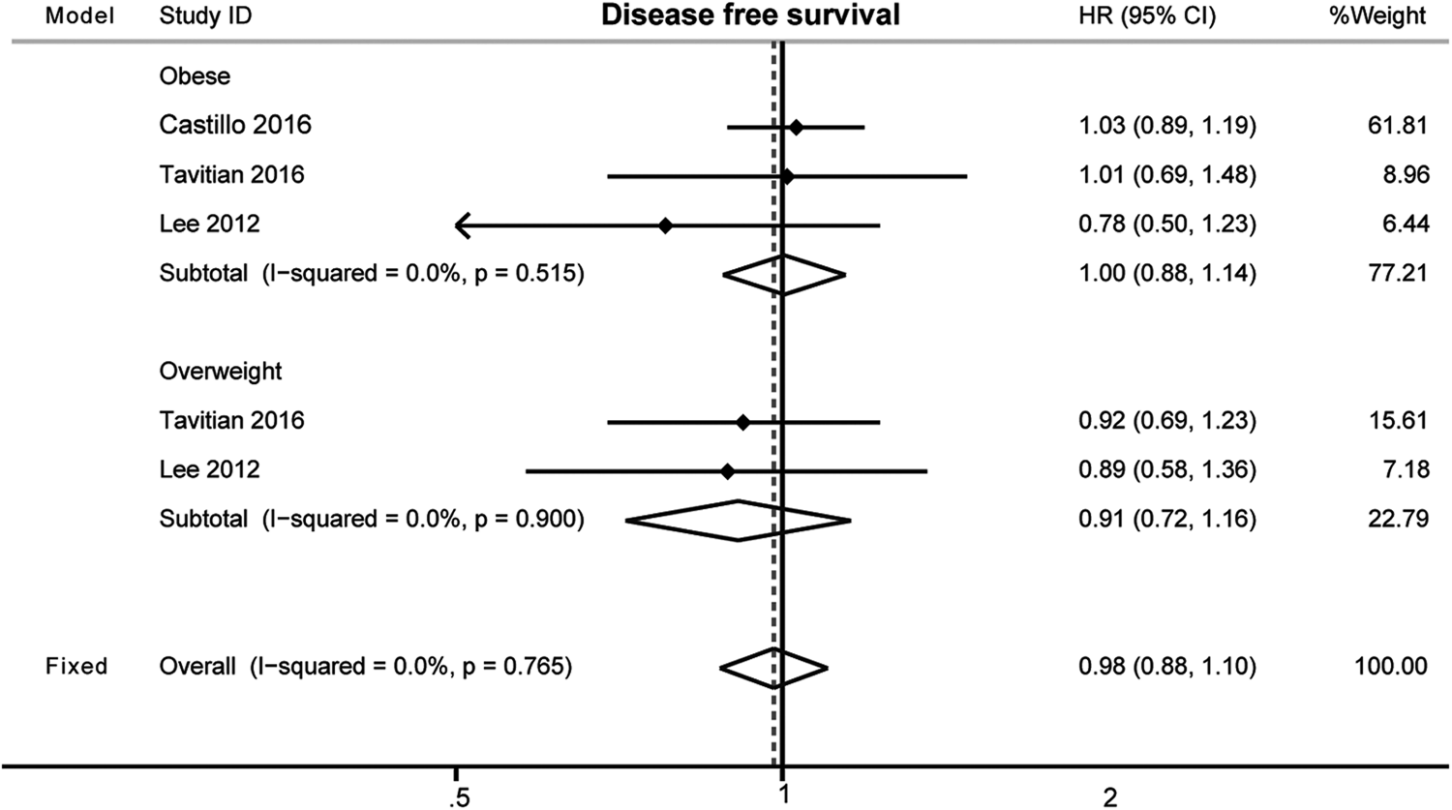
The effects of high BMI on the disease free survival of non-APL AML patients.

The HRs for disease free survival (DFS) were available in three studies about non-APL AML (Table 1). The results indicated that high BMI at diagnosis did not affect DFS of non-APL AML (HR, 0.98; 95% CI, 0.88–1.10; fixed effects, Fig. 4). The heterogeneity among studies was absent (P = 0.765; I^2^ = 0%). Specifically with respect to obesity, there was still no association between obesity and DFS (HR, 1.00; 95% CI, 0.88–1.14; P = 0.951; I^2^ = 0%; fixed effects, Fig. 4).

#### Significant association between high BMI and clinical outcome of APL

**Figure 5:**
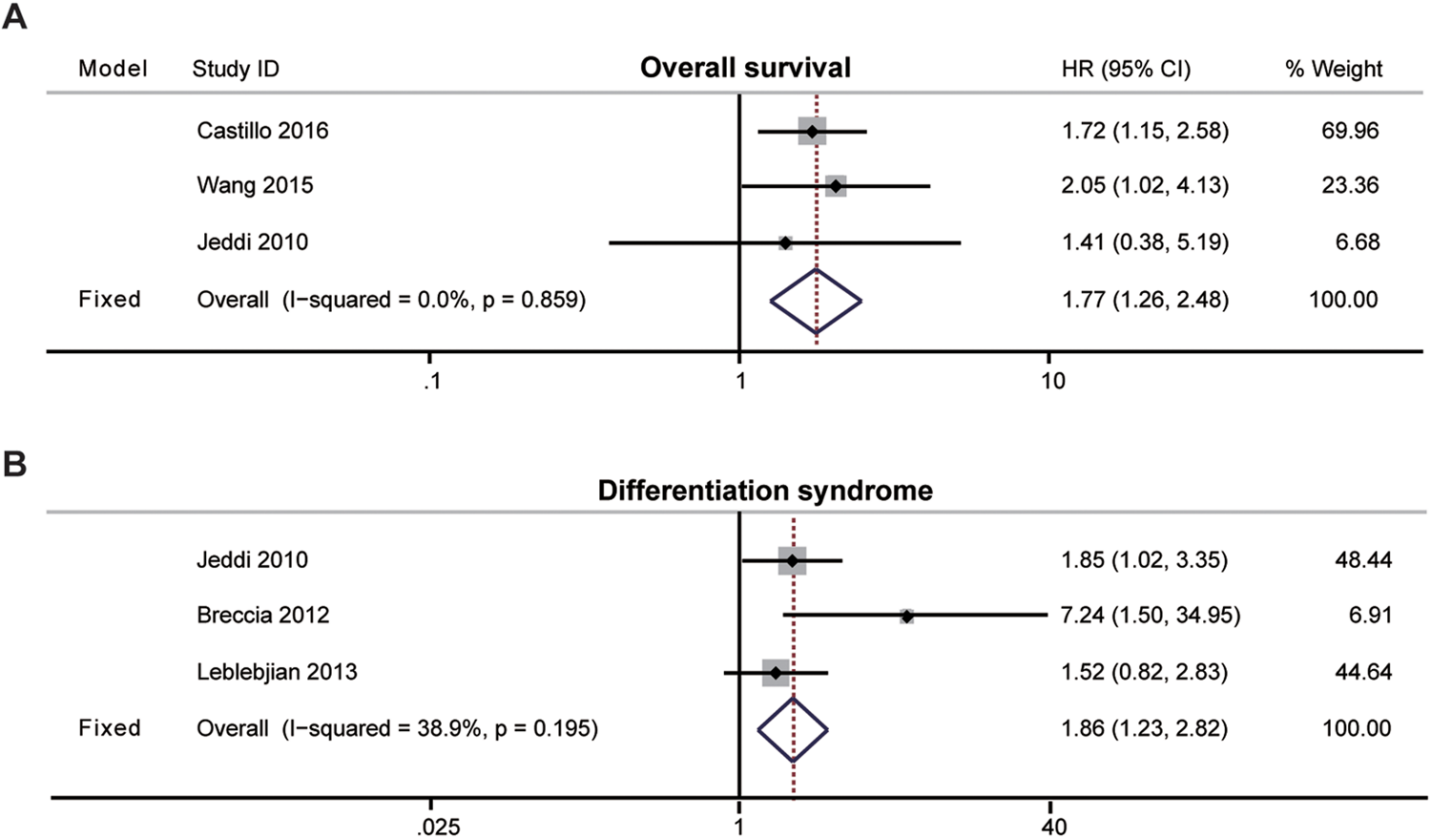
The effects of high BMI on the clinical outcome of APL patients. (**A**) Meta-analysis of the overall survival (OS) of APL according to BMI with the fixed- effects model. (**B**) Meta-analysis of the differentiation syndrome of APL according to BMI with the fixed-effects model.

APL is a unique subtype of AML. Surprisingly, we found that among patients with APL, those with high BMI had a shorter duration of OS than those with low BMI after pooling three relevant studies (HR, 1.77; 95% CI, 1.26–2.48; P = 0.001; I^2^ = 0%; fixed effects; Fig. 5A)^15,24,32^. Moreover, high BMI in patients with APL was associated with an increased risk of developing differentiation syndrome, a severe complication of chemotherapy, (HR, 1.86; 95% CI, 1.23–2.82; P = 0.003; I^2^ = 38.9%; fixed effects; Fig. 5B), according to three related studies^23,37,38^. The DFS data of APL was available in only one publication, which demonstrated that obese patients have worse DFS (HR, 1.53; 95% CI, 1.03–2.27, p = 0.04)^15^.

## Discussion

Increasing evidence has demonstrated that obesity is correlated to the incidence and clinical outcome of AML^5–7,29,39^. In our meta-analysis, we systematically collected related literature and analyzed the association between obesity and AML, including APL. First, we observed that both overweight and obesity increased the risk for AML. This is an important update since previous studies showed no correlation between overweight and incidence of AML^7,39,40^. Second, our study was the first to evaluate the relationship between obesity and the clinical outcomes (OS and DFS) of AML using a meta-analytical methodology. We found that obesity had no effect on the OS or DFS of non-APL AML, whereas overweight or obese patients with APL had a poorer OS and a higher risk of developing differentiation syndrome. Overall, obesity was closely related to AML, especially APL.

Exploring the association between obesity and AML is important for drug development and the battle against AMLs. However, mechanistic studies investigating the adverse association between obesity and AML are still in the early stages. Recently, epigenome-wide association studies identified that BMI is associated with widespread changes in DNA methylation^41^. In fact, fatty acid binding protein 4 (FABP4)/IL-6/STAT3/DNA methyltransferase 1 (DNMT1) have been shown to link obesity to the growth of AML cells^42^. Li *et al*. demonstrated that the obesity-associated gene FTO is highly expressed in AML with the MLL-fusion gene, thereby promoting leukemogenesis and inhibiting ATRA-induced cell differentiation^43^. Moreover, studies have shown that leukemia stem cells with a high expression of CD36 (fatty acid transporter) are prone to be chemoresistance^44^, and CD36^+^ patients with AML have a poorer leukemia-free survival^45^.

Apart from the mechanisms illustrated above, the function of obesity in contributing to leukemogenesis requires more research. Adipose tissue is a complex and active endocrine organ and can secrete various bioactive adipokines, hormones, inflammatory factors, angiogenesis factors, and free fatty acids. These secretory factors have been shown to participate in production and progression of tumors^46,47^, suggesting they may also contribute to the leukemogenesis of AML. Furthermore, as adipocytes are one of the most abundant cell types in blood-producing bone marrow^48^, direct cell-to-cell interaction cannot be disregarded, as Tabe *et al*. have demonstrated that the direct cell-to-cell interaction between adipocytes and APL cells is required for adipocyte-induced anti-apoptotic effect^49^. Also, it should be noted that obese individuals have unique pharmacokinetics compared to normal-weight controls. Reduced efficacy of chemotherapy drugs in overweight and obese patients, probably as a result of altered liver enzyme function, is a likely and often underestimated event related to cancer mortality^46^. It is also unclear whether the increased incidence and adverse outcome of obese patients with leukemia are associated to complications from obesity-related co-morbidities and a chronic inflammatory state or to obesity itself. In addition, microbiota can modulate the response to cancer therapy and susceptibility to toxic side effects, and obesity-induced gut microbial metabolites alterations may influence the clinical outcomes of AML patients^47^.

When evaluating the prognosis of patients with AML, the treatment strategies and patient’s characteristics should also be factors to consider, including gender, age, chromosome karyotype, genetic changes, and ECOG. In our meta-analysis, multivariate analysis was conducted to avoid the interference of confounding factors in most studies (Table 1 and Supplementary Table S2). Another important issue is that the dosing strategies may be different for the obese AML patients in different studies. For this, Percival *et al*. summarized several retrospective analyses and concluded that dosing according to body surface area based on ideal body weight (cap) or actual body weight (not cap) have similar complete remission rates, toxicity, and overall survival in obese patients with AML^50^.

Our finding that obesity has no effect on the OS of adult patients with AML was somewhat surprising, particularly because a high BMI is associated with poorer OS in pediatric patients with AML^51^. This discrepancy may be due to the physiological and metabolic differences between children and adults. However, we did find that obesity was associated with poorer OS in adult patients with APL. Actually, Tabe *et al*. showed that the leptin receptor is highly expressed on APL cells, and co-culturing of mesenchymal stem cell-derived adipocytes with APL cells significantly reduced ATRA and doxorubicin-induced apoptosis of APL cells^49^. More mechanistic studies are required to investigate the association between obesity and APL.

Our meta-analysis may carry inherent limitations within the published literature. First, there is risk for publication, ecological, and racial bias. Second, although the survival data included was primarily obtained through multivariable analyses considering the most influential confounder, some confounders may have still been ignored. Third, BMI is inappropriate for evaluating adiposity in individuals at extreme heights or older ages. Moreover, self-reported height and weight in some studies may be inaccurate and thus result in classification errors. Obese individuals vary in their distribution of visceral and subcutaneous fat. Since the function and metabolism between the two types are different, individuals who have the same BMI may respond differently to risk factors and cancer drugs due to variance in fat distributions. Finally, the research on APL is relatively limited because its incidence is lower than in other subtypes of AML. Further studies should be conducted to sufficiently address these issues.

In conclusion, both overweight and obesity were associated with increased risk of AML, with obesity producing more palpable effects. However, obesity does not significantly affect the OS and DFS of non-APL AML. APL patients are, more obese, on average, than those with other forms of AML, and obesity is an adverse prognostic factor for clinical outcome in APL (demonstrated by reduced OS and the increased incidence of differentiation syndrome). Thus, APL is more specifically associated with obesity.

Evaluating the underlying mechanisms responsible for their relationship is necessary to develop the novel therapeutic approaches for AML. Also, given that high BMI increases the incidence of AML, controlling body weight may be considered for obese individuals. In addition, for obese APL patients, more attention should be given to possible ATRA-associated complications during induction therapy.

## Electronic supplementary material

Supplementary Information
