# Supplementary material for: Influence of body mass index on incidence and prognosis of acute myeloid leukemia and acute promyelocytic leukemia: A meta-analysis
Source: Sci Rep. 2017 Dec 21;7:17998. doi: 10.1038/s41598-017-18278-x (PMC5740068; doi:10.1038/s41598-017-18278-x)
Supplement: Supplementary file 1 — Supplementary Information [file 41598_2017_18278_MOESM1_ESM.pdf]

Influence of body mass index on incidence and prognosis of acute myeloid leukemia and acute promyelocytic leukemia: A meta-analysis

Shufen Li[1,*], Li Chen[2,*], Wen Jin[1,2], Xuefei Ma[1], Yunlin Ma[3], Fangyi Dong[2], Hongming Zhu[2], Junmin Li[2], Kankan Wang[1,*]

1. State Key Laboratory of Medical Genomics and Shanghai Institute of Hematology, Ruijin Hospital, Shanghai Jiao Tong University School of Medicine, Shanghai 200025, China
2. Department of Hematology, Ruijin Hospital, Shanghai Jiao Tong University School of Medicine, Shanghai 200025, China
3. State Key Laboratory of Medical Genomics, Institute of Health Sciences, Shanghai Institutes for Biological Sciences, Chinese Academy of Sciences & Shanghai Jiao Tong University School of Medicine, University of Chinese Academy of Sciences, Shanghai, China.

*. These authors contributed equally to this work.

Correspondence: Kankan Wang, State Key Laboratory of Medical Genomics and Shanghai Institute of Hematology, Ruijin Hospital, Shanghai Jiao Tong University of Medicine, 197 Ruijin Er Rd, Shanghai 200025, China; e-mail: kankanwang@shsmu.edu.cn; Tel: 86-21-64370045 ext 611109; Fax: 86-21-64743206.

A

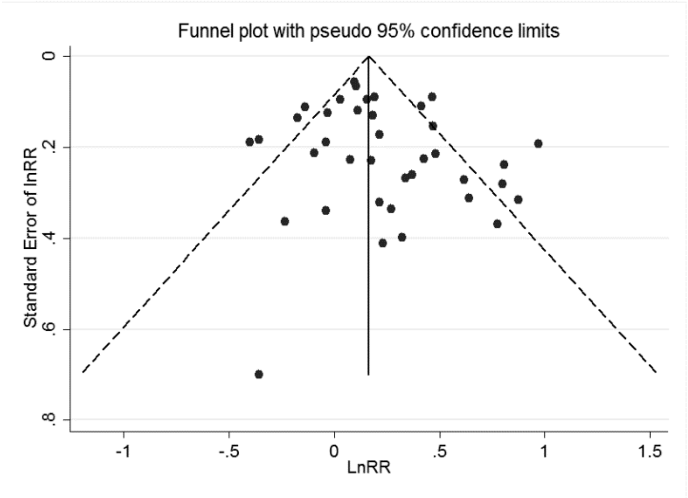

B

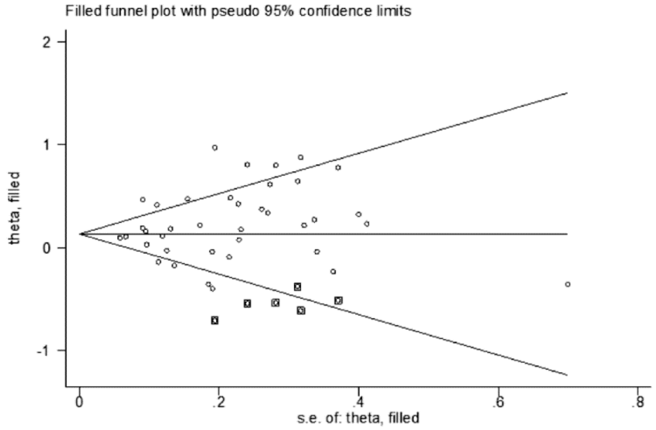

C

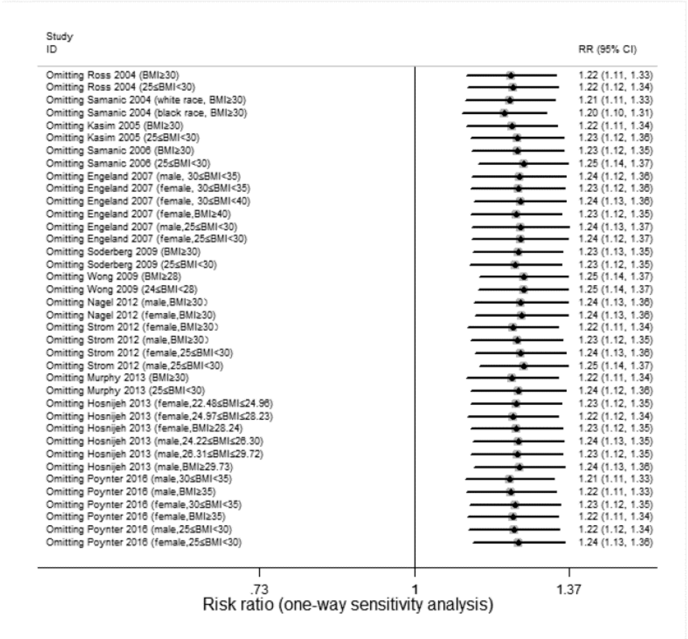

Supplementary Figure 1. Supporting data for the association between BMI and relative risk of AML. (**A**) Assessment of publication bias by funnel plots. (**B**) Identification of potential missing studies by the trim and fill analysis. (**C**) Confirmation of the stability of the pooled results by one-way sensitivity analysis.

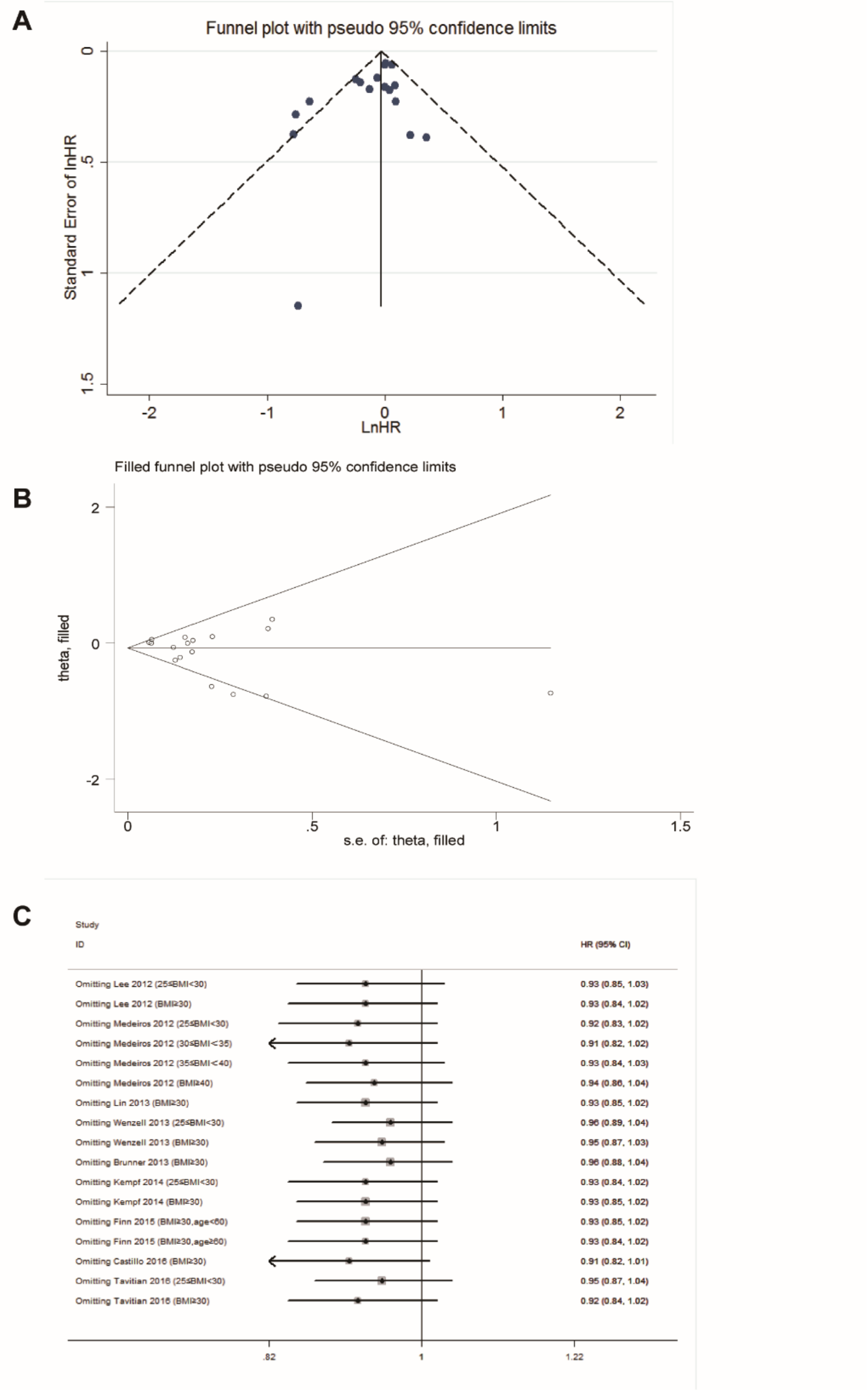

Supplementary Figure 2. Supporting data for the association between BMI and overall survival of AML. (**A**) Assessment of publication bias by funnel plots. (**B**) Identification of potential missing studies by the trim and fill analysis. (**C**) Confirmation of the stability of the pooled results by one-way sensitivity analysis.

Study	Quality indicators from the Newcastle-Ottawa scale			Score
	Selection	Comparability	Exposure or Outcome	
**Studies on relative risk**				
Samanic 2004	3	2	3	8
Ross 2004	3	2	3	8
Kasim 2005	3	2	2	7
Samanic 2006	4	2	3	9
Engeland 2007	4	2	3	9
Wong 2009	3	2	1	6
Soderberg 2009	3	2	3	8
Strom 2012	4	2	2	8
Nagel 2012	3	2	3	8
Murphy 2013	3	2	3	8
Hosnijeh 2013	4	2	3	9
Poynter 2016	4	2	2	8
**Studies on overall survival**				
Jeddi 2010	2	2	3	7
Lee 2012	3	2	3	8
Medeiros 2012	3	2	3	8
Lin 2013	3	2	3	8
Wenzell 2013	2	2	3	7
Brunner 2013	3	2	3	8
Kempf 2014	3	2	3	8
Wang 2015	2	0	3	5
Finn 2015	2	2	3	7
Castillo 2016	3	2	3	8
Tavitian 2016	3	2	3	8
**Studies on differentiation syndrome**				
Jeddi 2010	2	2	3	7
Breccia 2012	2	2	3	7
Leblebjian 2013	2	2	3	7

Supplementary Table S1: Assessment of study quality.

**First author, year**	**Treatment protocol**	**Analysis of treatment for multiple factors**
**Studies about obesity and survival (AML)**		
Castillo 2016	CALGB9621,10503,19808 protocol	CALGB9621 vs 10503 vs 19808
Tavitian 2016	Induction：3+7(DA/IA) Consolidation：Data not given	AlloSCT vs Non-alloSCT
Kempf 2014	AML10, AML12, AML13 protocol	Not available
Lin 2013*	Induction：3+7(DA/IA) Consolidation：Data not given	Not available
Lee 2012	Induction：HiDAC+IDA Consolidation：Varied over time	Treatment decade
Medeiros 2012	SWOG (Trial S8600, S9031,S9126,S9333,S9500, S9617, S9918, and S0106) protocol	Not available
Finn 2015	Intensive and Non-intensive chemotherapy	Intensive vs Non-intensive chemotherapy
Wenzell 2013	Induction：3+7（IA/DA/MA）Consolidation：HiDAC or the scheme containing Ara-c or Auto/alloSCT	Not available
Brunner 2013	Induction：Mainly for 3+7（IA/DA）Consolidation：Data not given	Chemotherapy Dosing
**Studies about obesity and survival (APL)**		
Jeddi 2010	PETHEMA LPA99 protocol	Same scheme
Castillo 2016	C9710 protocol	Same scheme
Wang 2015#	Induction：ATRA+HHT，Consolidation：ATRA+HHT+Ara-c；Maintenance：ATRA+MTX+6-MP	Same scheme
Breccia 2012	GIMEMA AIDA-0493 and AIDA-2000 protocol	Not available
**Studies about obesity and Differentiation Syndrome**		
Jeddi 2010	PETHEMA LPA99 protocol	Same induction scheme
Breccia 2012	GIMEMA AIDA-0493 and AIDA-2000 protocol	Same induction scheme
Leblebjian 2013	ATRA+CT; ATRA+ATO; ATRA+GO; ATRA+ATO+GO; ATRA+Hu	Different induction schemes were compared

Supplementary Table S2: The treatment strategy in the eligible paper included in the analysis of the prognosis value of obesity in AML. overweight: BMI⩾25 Obese：BMI⩾30；*Obese：BMI ⩾130% ideal body weight；#overweight: BMI⩾23. DA: Doxorubicin+Cytarabine. IA: Idarubicin+Cytarabine. HiDAC: High dose cytarabine. IDA: Idarubicin. MA: Mitoxantrone+Cytarabine. Ara-c: Cytarabine. MTX: Methotrexate. HHT: Homoharringtonine. ATRA: All-trans-retinoic acid. 6-MP: Mercaptopurine. CT: Chemotherapy. ATO: Arsenic trioxide. GO: Gemtuzumab ozogamicin. Hu: Hydroxyurea. AlloSCT :Allogeneic stem cell transplantation. AutoSCT: Autologous stem cell transplantation.
